# Exploring the ubiquitination regulatory network: opening new perspectives for rheumatoid arthritis therapy

**DOI:** 10.1080/07853890.2025.2600171

**Published:** 2025-12-09

**Authors:** Fanfan Wang, Jian Liu, Jianting Wen, Yue Sun, Mingyu He

**Affiliations:** a The First Affiliated Hospital of Anhui University of Chinese Medicine, Hefei, Anhui, China; b Department of Rheumatism Immunity, The First Affiliated Hospital of Anhui University of Chinese Medicine, Hefei, Anhui, China

**Keywords:** Rheumatoid arthritis, ubiquitination, expression, mechanism, therapy

## Abstract

Rheumatoid arthritis (RA) is a complex autoimmune disorder characterized by chronic systemic inflammation, primarily affecting the joints and synovium, but also involving multiple extra-articular tissues. This persistent inflammation contributes to progressive joint damage and the development of various systemic comorbidities. Despite significant advances in RA research, the underlying pathogenic mechanisms remain incompletely elucidated. Ubiquitination, a critical post-translational modification, regulates protein stability, localization, and function, and is essential in numerous biological processes, including cell signaling, immune regulation, and inflammatory responses. Emerging evidence highlights the pivotal role of ubiquitination in the pathogenesis of RA. This review provides a comprehensive overview of recent advances in the study of ubiquitination in RA, delineates its mechanistic contributions to disease development, and evaluates the therapeutic potential of targeting ubiquitination pathways. By offering new insights into RA pathophysiology, this review lays a theoretical and experimental foundation for the development of more effective, targeted therapeutic strategies, potentially opening new avenues for RA treatment.

## 1. Introduction

Rheumatoid arthritis (RA) is a systemic autoimmune disease marked by chronic, progressive, and destructive inflammation of the joints [1,2]. Affecting ∼1% of the global population, RA is typically diagnosed between the ages of 40 and 50 and shows a significantly higher prevalence in women, with an incidence rate three to five times greater than that in men [3,4]. The primary therapeutic goals in RA management include pain relief, inflammation control, immunosuppression, cartilage preservation, and delaying disease progression [5]. Commonly used pharmacologic treatments encompass non-steroidal anti-inflammatory drugs (NSAIDs), glucocorticoids, and disease-modifying anti-rheumatic drugs (DMARDs). Despite advances in pharmacotherapy, a substantial proportion of patients fail to achieve sustained remission [6]. Consequently, there is a pressing need for novel therapeutic strategies that can selectively target pathogenic cells or molecular pathways driving joint inflammation and destruction, while minimizing reliance on broad-spectrum immunosuppressants. Such targeted approaches hold promise for improving disease outcomes and enhancing the long-term management of RA.

Ubiquitination is a fundamental cellular process that regulates protein activity, stability, and protein-protein interactions by modulating the activation and inactivation of target proteins [7,8]. This post-translational modification involves the covalent attachment of one or more ubiquitin molecules to substrate proteins, a reaction catalyzed sequentially by three classes of enzymes: E1 ubiquitin-activating enzymes, E2 ubiquitin-conjugating enzymes, and E3 ubiquitin ligases. During this process, the C-terminal glycine of ubiquitin is conjugated to the lysine residues of target proteins, resulting in either monoubiquitination or the formation of polyubiquitin chains with distinct topologies and functional outcomes [9,10]. Ubiquitination is dynamically regulated by deubiquitinating enzymes (DUBs), which cleave ubiquitin moieties from substrates, prevent polyubiquitin chain elongation, and recycle ubiquitin from precursors or degradation-targeted proteins [11,12]. The precise balance between ubiquitination and deubiquitination is essential for maintaining protein homeostasis and modulating key cellular processes.

Recent studies have highlighted the pivotal role of ubiquitination—a critical post-translational modification—in the pathogenesis of RA. For example, myeloid cell-specific deletion of the deubiquitinase A20 results in dysregulated NF-κB signaling, a central pathway in RA-associated inflammation, leading to spontaneous arthritis development [13]. Moreover, ubiquitination of DPP4 by the E3 ubiquitin ligase Mid1 has been shown to enhance synoviocyte proliferation and invasiveness, thereby aggravating synovitis in RA [14]. This review presents the first comprehensive and systematic summary of the multi-layered regulatory network of ubiquitination modifications in RA, focusing on the aberrant expression and functional roles of ubiquitination-related enzymes in RA progression. Building upon existing literature, we integrate mechanistic insights into the molecular functions of key ubiquitination enzymes with their clinical implications, and explore emerging therapeutic strategies targeting ubiquitination pathways. These findings are expected to provide valuable perspectives for elucidating RA pathogenesis and identifying novel therapeutic targets.

## 2. Ubiquitination

Ubiquitin is a highly conserved, ubiquitously expressed protein found in all eukaryotic cells. Comprising 76 amino acids and with an approximate molecular weight of 8.5 kDa, ubiquitin plays a fundamental role in tagging proteins for degradation. Its C-terminus contains two glycine residues, which are functionally essential for covalent conjugation to substrate proteins [15]. Additionally, ubiquitin harbors seven lysine residues (K6, K11, K27, K29, K33, K48, K63) and one N-terminal methionine (M1) [16]. These residues enable the formation of diverse ubiquitin chain linkages and topological structures, which in turn dictate the fate and functional outcomes of modified substrate proteins [15].

Ubiquitination is a post-translational modification (PTM) in which ubiquitin is covalently attached to specific lysine residues of target proteins. This modification is central to the regulation of protein degradation and cellular homeostasis. It is estimated that ∼90% of proteins undergo ubiquitination to perform their cellular functions [17]. The ubiquitination cascade is mediated by a series of enzymatic steps involving three major classes of enzymes: E1 ubiquitin-activating enzymes, E2 ubiquitin-conjugating enzymes, and E3 ubiquitin ligases. In an ATP-dependent manner, E1 enzymes activate and transfer ubiquitin to E2 enzymes. Subsequently, E2 enzymes, in coordination with E3 ligases, facilitate the transfer of ubiquitin to the substrate protein [18]. Among these, E3 ligases play a central role by recognizing specific substrate proteins and determining substrate specificity [10].

To date, two E1 enzymes and ∼40 E2 enzymes have been identified in humans [16]. In contrast, more than 800 E3 ligases have been characterized in mammals [19]. Based on their structural domains and mechanisms of action, E3 ligases are classified into three major families: (1) HECT (Homologous to E6-AP Carboxyl Terminus) ligases, which form a transient thioester bond with ubiquitin before transferring it to substrates; (2) RING (Really Interesting New Gene) finger ligases, which act as scaffolds to facilitate direct ubiquitin transfer from E2 to substrate; and (3) RBR (RING-between-RING) ligases, which incorporate features of both HECT and RING ligases to mediate ubiquitination [15,20].

## 3. Alterations in the expression of ubiquitination-related enzymes in RA

**Figure 1.**
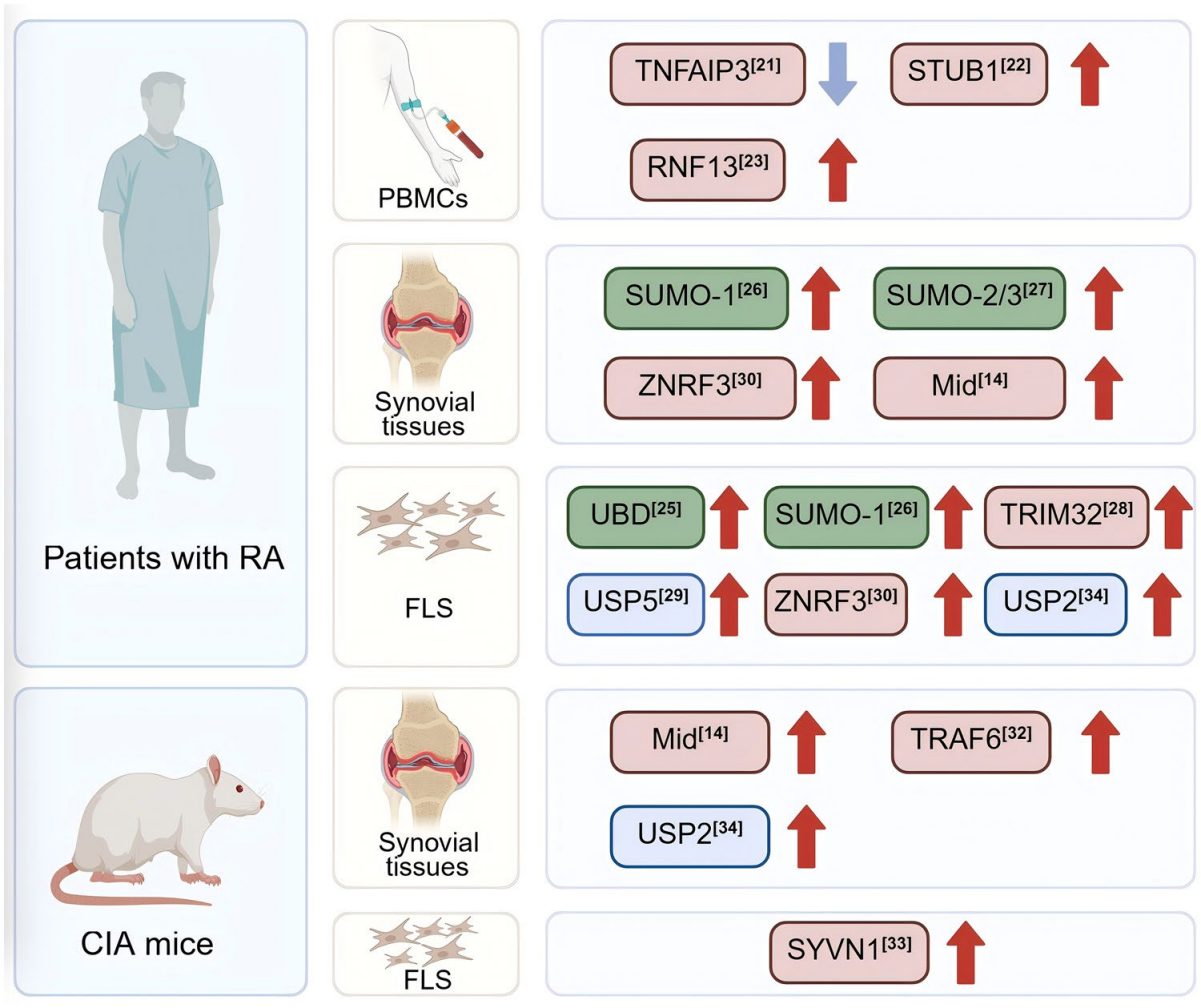
Alterations in the expression of ubiquitination-related enzymes in RA patients. Enzymes are color-coded by category: Red (E3 ligases); Blue (DUBs); Green (other ubiquitination-associated factors).

Recent studies provide new evidence on changes in ubiquitination-related enzyme levels in RA patients and *in vivo* models, as illustrated in Figure 1. These primarily involve E3 ubiquitin ligases, deubiquitinating enzymes (DUBs), and SUMOylation-related enzymes. Their dysregulated expression patterns are detailed below.

### 3.1. Dysregulated expression of E3 ubiquitin ligases

Recent studies have provided accumulating evidence of altered expression patterns of ubiquitination-related enzymes in both RA patients and *in vivo* models, as illustrated in Figure 1. For instance, one study reported a significant downregulation of mRNA expression of the deubiquitinating enzyme *TNFAIP3* in peripheral blood mononuclear cells (PBMCs) from RA patients compared to healthy controls [21]. Conversely, elevated levels of the E3 ubiquitin ligase STUB1 were detected in CD4^+^ T cells derived from RA patients, with distinct expression patterns observed in Th17 and Treg cell subsets [22]. Using high-content screening, another study identified the lysosome-associated E3 ligase RNF13 as a positive regulator of Toll-like receptor (TLR)-mediated innate immune responses, and further demonstrated its increased expression in PBMCs from RA patients [23].

Fibroblast-like synoviocytes (FLSs), key effector cells in RA pathogenesis, contribute to persistent synovial inflammation and joint destruction [24]. The expression of the E3 ubiquitin ligase TRIM32 is also markedly upregulated at both mRNA and protein levels in RA-FLSs compared to OA-FLSs. Stimulation with TNF-α further induced time-dependent increases in TRIM32 expression, suggesting a link between inflammatory signaling and TRIM32 activity in RA pathophysiology [25]. Additionally, expression of the E3 ligase ZNRF3 is significantly elevated in synovial tissues and FLSs from RA patients when compared to normal controls derived from trauma patients [26].

The collagen-induced arthritis (CIA) model remains a widely used experimental system for studying autoimmune mechanisms in RA [27]. Analysis of publicly available GEO datasets revealed upregulation of the E3 ligase *Mid1* in human RA synovial tissues, a finding further validated in CIA mice [14]. In CIA rats, both mRNA and protein levels of the E3 ligase *TRAF6* were significantly increased compared to controls (*p* < 0.05). Following lentiviral-mediated *TRAF6* knockdown, a marked reduction in *TRAF6* expression was observed, supporting its role in RA pathogenesis [28]. Moreover, the expression of the E3 ligase Synoviolin (SYVN1) was found to be elevated ∼3-fold in synovial fibroblasts from CIA rats compared to normal fibroblasts [29].

### 3.2. Dysregulated expression of deubiquitinating enzymes (DUBs)

DUBs participate in RA pathology by regulating protein deubiquitination. The deubiquitinating enzyme USP5 shows higher expression in RA-FLSs relative to OA-FLSs, with IL-1β stimulation inducing a similar time-dependent increase [30]. Both *in vivo* (CIA rat model) and *in vitro* (human RA-FLSs) studies demonstrated increased expression of the deubiquitinating enzyme USP2, further implicating its involvement in RA progression [31].

### 3.3. Dysregulated expression of other ubiquitination-associated factors

In RA synovial tissues, proteins containing ubiquitin-binding domains (UBDs) are significantly overexpressed compared to healthy controls [32]. Similarly, members of the small ubiquitin-like modifier (SUMO) family, particularly SUMO-1, show markedly increased expression in FLSs and synovial tissues (STs) from RA patients [33]. Furthermore, SUMO-2 and SUMO-3 expression levels are significantly elevated in both RA tissues and RA-derived FLSs relative to those from osteoarthritis (OA) patients [34].

## 4. Correlation between the expression of ubiquitination-related enzymes and clinical disease activity and inflammatory cytokines in RA

**Figure 2.**
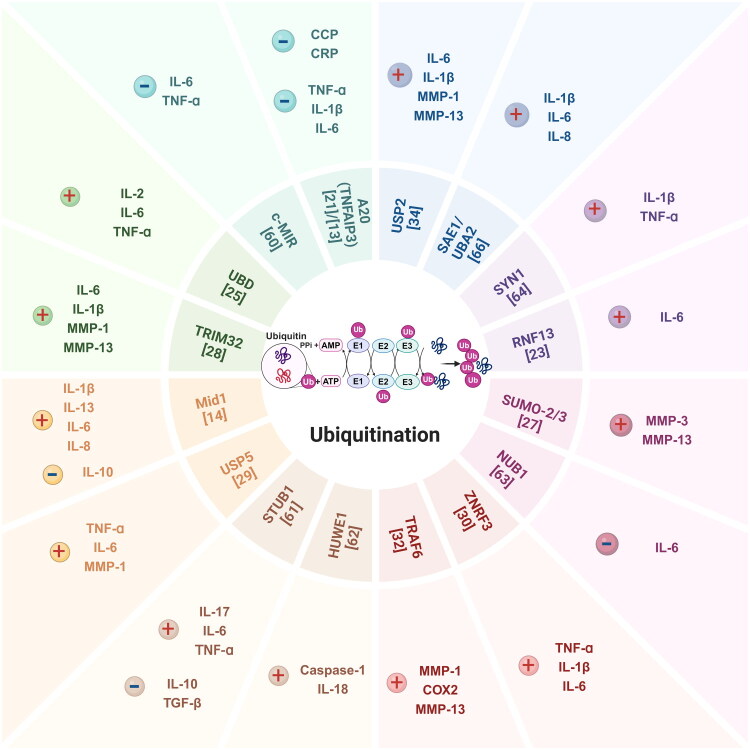
Correlation between the expression of ubiquitination-related enzymes and clinical disease activity, as well as inflammatory cytokines. *Note:* ‘+’ Indicates a positive correlation, while ‘−’ indicates a negative correlation.

This section first outlines key clinical parameters used to assess disease activity, including C-reactive protein (CRP), erythrocyte sedimentation rate (ESR), and anti-cyclic citrullinated peptide (anti-CCP) antibodies, as well as central inflammatory and effector cytokines such as tumor necrosis factor-α (TNF-α), interleukin-1β (IL-1β), interleukin-6 (IL-6), and matrix metalloproteinases (MMPs). Subsequently, we present an integrated analysis of current evidence correlating the expression of ubiquitination-related enzymes with these critical clinical and immunological markers, which collectively define RA disease activity status (Table 1 and Figure 2).

**Table 1.** Correlation between the expression of ubiquitination-related enzymes and clinical disease activity and inflammatory cytokines in RA.

Category	Marker	A20 (TNFAIP3)	c-MIR	UBD	TRIM32	Mid1	USP5	STUB1	HUWE1	TRAF6	ZNRF3	NUB1	SUMO-2/3	RNF-13	SYN1	SAE1/UBA2	USP2
Clinical biomarkers	CRP	−	NA	NA	NA	NA	NA	NA	NA	NA	NA	NA	NA	NA	NA	NA	NA
	CCP	−	NA	NA	NA	NA	NA	NA	NA	NA	NA	NA	NA	NA	NA	NA	NA
Pro-inflammatory cytokines	TNF-α	−	−	+	NA	NA	+	+	NA	NA	+	NA	NA	NA	+	NA	NA
	IL-1β	−	NA	NA	+	+	NA	NA	NA	NA	+	NA	NA	NA	+	+	+
	IL-2	NA	NA	+	NA	NA	NA	NA	NA	NA	NA	NA	NA	NA	NA	NA	NA
	IL-6	−	−	+	+	+	+	+	NA	NA	+	−	NA	+	NA	+	+
	IL-8	NA	NA	NA	NA	+	NA	NA	NA	NA	NA	NA	NA	NA	NA	+	NA
	IL-13	NA	NA	NA	NA	+	NA	NA	NA	NA	NA	NA	NA	NA	NA	NA	NA
	IL-17	NA	NA	NA	NA	NA	NA	+	NA	NA	NA	NA	NA	NA	NA	NA	NA
	IL-18	NA	NA	NA	NA	NA	NA	NA	+	NA	NA	NA	NA	NA	NA	NA	NA
Anti-inflammatory cytokines	IL-10	NA	NA	NA	NA	−	NA	−	NA	NA	NA	NA	NA	NA	NA	NA	NA
	TGF-β	NA	NA	NA	NA	NA	NA	−	NA	NA	NA	NA	NA	NA	NA	NA	NA
Other inflammatory mediators	COX2	NA	NA	NA	NA	NA	NA	NA	NA	+	NA	NA	NA	NA	NA	NA	NA
	MMP-1	NA	NA	NA	+	NA	+	NA	NA	+	NA	NA	NA	NA	NA	NA	+
	MMP-3	NA	NA	NA	NA	NA	NA	NA	NA	NA	NA	NA	+	NA	NA	NA	NA
	MMP-13	NA	NA	NA	+	NA	NA	NA	NA	+	NA	NA	+	NA	NA	NA	+
	Caspase-1	NA	NA	NA	NA	NA	NA	NA	+	NA	NA	NA	NA	NA	NA	NA	NA

*Note:* ‘+’ = Positive correlation; ‘−’ = Negative correlation.

‘NA’ = No available research data (correlation not assessed in existing studies).

RA disease activity is commonly evaluated using a combination of clinical and laboratory indicators, particularly ESR and CRP levels [35]. Additionally, rheumatoid factor (RF) and anti-CCP antibodies also represent important serological biomarkers for diagnosis and prognostic stratification of disease activity [36,37].

Inflammatory cytokines play central roles in RA pathogenesis. TNF-α is a key pro-inflammatory mediator that activates leukocytes and endothelial cells, driving the production of downstream cytokines and chemokines [38]. IL-1β contributes to synovial hyperplasia, fibrosis, and cartilage degradation in RA joints [39]. IL-6, a multifunctional cytokine secreted by a variety of immune and stromal cells, together with IL-8, promotes leukocyte recruitment and perpetuates inflammation [40]. IL-17 further amplifies synovial inflammation and stimulates angiogenesis within the joint microenvironment [41]. Notably, IL-6 can act synergistically with TNF-α and IL-1β to exacerbate synovial inflammation and promote chronic disease progression [42]. In contrast, anti-inflammatory cytokines such as IL-10 and TGF-β play immunoregulatory roles; IL-10, for instance, can suppress the production of pro-inflammatory cytokines in RA synovial tissue [43]. Previous studies have shown that cytokine imbalances—including IL-10, IL-6, IL-4, and IL-17—are associated with aberrant activation of the NF-κB pathway in active RA, contributing to systemic inflammation and a prothrombotic state [44]. IL-13, a Th2 cytokine, is also implicated in RA pathogenesis; its serum levels are elevated in early RA and positively correlate with disease activity scores [45,46]. Additionally, IL-18 promotes the production of TNF-α and IL-1β, thereby intensifying the inflammatory response [47]. Other key inflammatory mediators, such as cyclooxygenase-2 (COX-2), NF-κB, and TNF-α, are involved in the recruitment and activation of immune cells at sites of chronic inflammation [48].

In RA, MMPs produced by synovial tissue are key mediators of cartilage and bone destruction [49,50]. These enzymes degrade various components of the extracellular matrix, with collagenases (MMP-1, MMP-13) and stromelysins (particularly MMP-3) playing central roles in RA pathophysiology [51–55]. Their synthesis and activation are driven by pro-inflammatory cytokines and growth factors, with activated RA-FLSs being the primary source of MMPs and inflammatory mediators.

Caspase-1 is an essential inflammatory caspase responsible for cleaving and activating pro-inflammatory cytokines such as IL-1β and IL-18 [56]. In RA, elevated levels of these cytokines in synovial fluid contribute to persistent inflammation and joint destruction. The activation of caspase-1 and the assembly of inflammasomes are critical events that amplify innate immune responses and promote autoimmune-driven joint damage [57–59].

The expression of the A20 (also known as *TNFAIP3*) gene in PBMCs of RA patients is negatively correlated with anti-CCP antibody titers and CRP levels [21]. Beyond this clinical correlation, A20 is mechanistically involved in the regulation of inflammatory pathways. In A20-deficient mice, elevated production of TNF-α, IL-1β, and IL-6, along with NF-κB hyperactivation, leads to multi-organ autoimmunity and erosive polyarthritis resembling RA [13]. Moreover, gene transfer of c-MIR has been shown to suppress IL-6 production by synovial fibroblasts upon stimulation with TNF-α or IL-1β. Similarly, bone marrow-derived macrophages and dendritic cells (DCs) from c-MIR transgenic mice exhibit impaired IL-6 and TNF-α production in response to LPS stimulation [60].

The ubiquitin-binding protein UBD promotes the secretion of inflammatory cytokines such as IL-2, IL-6, and TNF-α [32]. Overexpression of TRIM32 in RA-FLSs enhances the expression of IL-6, IL-1β, MMP-1, and MMP-13 in response to TNF-α stimulation [25]. Knockdown of *Mid1* in MH7A cells reduces mRNA and protein levels of IL-1β, IL-13, IL-6, and IL-8, while increasing IL-10 expression [14]. Similarly, USP5 promotes the production of TNF-α, IL-6, and MMP-1, with increased secretion observed in USP5-overexpressing RA-FLSs following IL-1β stimulation [30]. Upregulation of STUB1 suppresses anti-inflammatory cytokines IL-10 and TGF-β, while significantly increasing IL-17A, IL-6, and TNF-α levels [61].

Furthermore, anti-HUWE1 antibody levels in plasma are positively correlated with caspase-1 and IL-18 concentrations [62]. Silencing of *TRAF6* significantly reduces the mRNA and protein expression of MMP-1, COX-2, and MMP-13 in RA models [28]. In mouse models of RA, *ZNRF3* silencing mitigates knee joint damage and lowers levels of TNF-α, IL-1β, and IL-6 [26]. Overexpression of *NUB1* inhibits NF-κB nuclear translocation and reduces IL-6 mRNA expression in IL-1β-stimulated RA-FLSs [63]. *In vitro* experiments demonstrate that TNF-α induces SUMO-2, but not SUMO-3, expression. Silencing of SUMO-2/3 results in increased expression of MMP-3 and MMP-13 upon TNF-α and IL-1β stimulation, along with elevated NF-κB activity. Pharmacological inhibition of NF-κB attenuates this induction [34]. Mice deficient in *RNF13* show resistance to endosomal TLR ligands and bacterial challenge, producing significantly lower levels of IL-6 in serum and tissues compared to wild-type mice [23]. TNF-α and IL-1β upregulate Synoviolin (SYVN1) expression through the Erk1/ETS1 signaling pathway, contributing to synovial hyperplasia in RA [64]. In RA patients, the presence of RF is significantly associated with polymorphisms in *TNFAIP3* [65]. In cells stimulated with TNF-α, IL-1β, IL-17, or LPS, the expression of SAE1 and UBA2 is upregulated. Knockdown of SAE1/UBA2 or treatment with geldanamycin (GA) reduces TNF-α-induced expression of IL-1β, IL-6, and IL-8 [66]. In HFLS-RA cells, overexpression of USP2 enhances the production of IL-6, IL-1β, MMP-1, and MMP-13, suggesting that USP2 upregulation promotes both inflammation and FLS proliferation [31].

## 5. Role and potential mechanisms of ubiquitination-related enzymes in RA

### 5.1. A20 and RA

**Figure 3.**
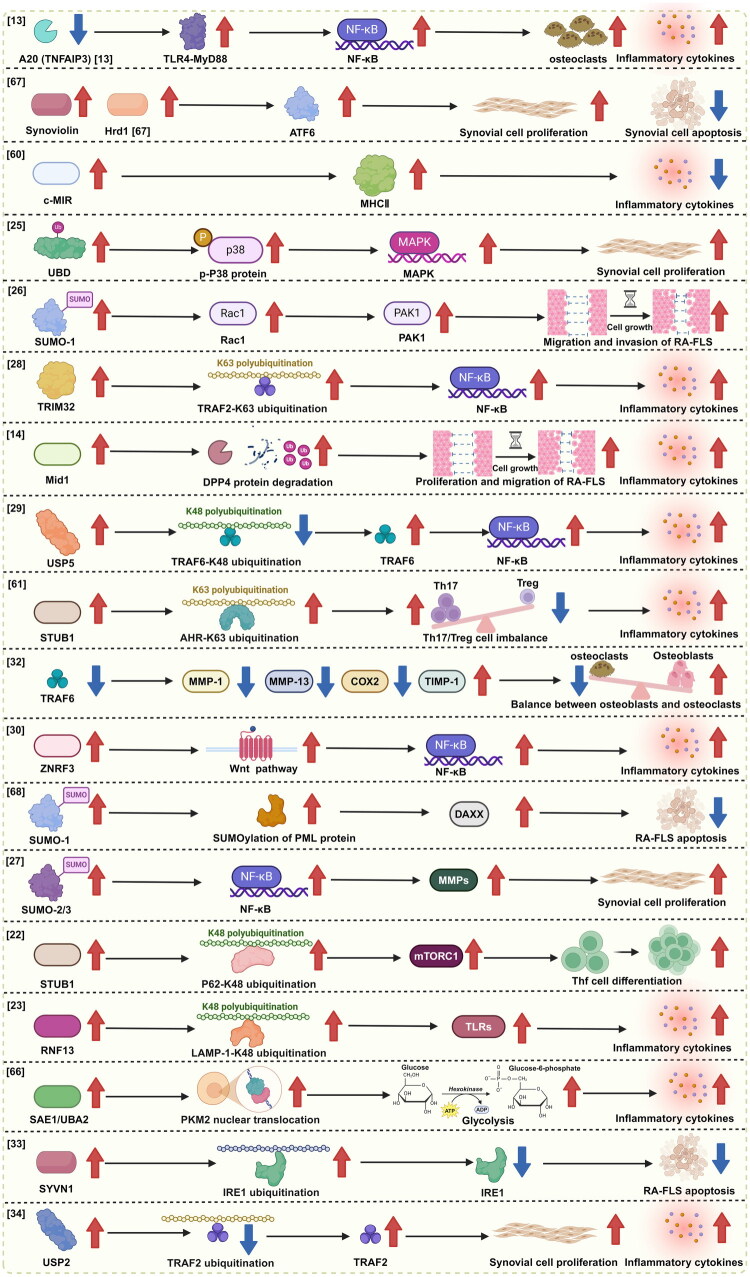
The potential of ubiquitination-related enzymes as targets and mechanisms in rheumatoid arthritis.

Studies utilizing bone marrow-specific *A20* (*TNFAIP3*) knockout mice have demonstrated that targeted deletion of *TNFAIP3* in hematopoietic cells leads to spontaneous development of severe, destructive polyarthritis, closely resembling the clinical and pathological features of RA. These mice exhibit markedly elevated levels of systemic inflammatory cytokines, sustained activation of the NF-κB pathway, and increased TNF production by macrophages. Further investigations have revealed that this arthritis phenotype in myeloid-specific *A20* knockout mice is dependent on TLR4-MyD88 and IL-6 signaling, but notably independent of TNF. In addition, a deficiency of A20 in myeloid cells enhances osteoclastogenesis (Table 2 and Figure 3). Collectively, these findings highlight the cell-specific regulatory functions of A20 in RA pathogenesis and support the development of A20-targeting strategies as a potential approach for cellular-level therapy in RA [13] (Table 2 and Figure 3).

**Table 2.** Roles and possible mechanisms of ubiquitination-related enzymes in RA.

Functional category	Ubiquitination-related enzymes	Targets	Rationale	Study outcome	References
E3 ubiquitin ligases	A20 (TNFAIP3)	TLR4-MyD88	Negative regulator of NF-ĸB signaling	Destructive polyarthritis in myeloid A20 knockout mice is TLR4-MyD88 and IL-6 dependent	[13]
	Synoviolin/Hrd1	ATF6	Activation factors of ATF6	Synoviolin/Hrd1 triggers synovial cell growth through its anti-apoptotic effect	[67]
	c-MIR	MHC II	Negative regulatory factors of antigen presentation in synovial tissue	C-MIR gene transfer inhibits IL-6 production in synovial fibroblasts stimulated with TNF-α or IL-1 β	[60]
	TRIM32	TRAF2	Positive regulatory factors of NF-κB signaling pathway	TRIM32 promotes the inflammatory response of fibroblast-like synovial cells in rheumatoid arthritis	[25]
	Mid1	DPP4	Positive regulatory factors for DPP4 proteasome degradation	Mid1 ubiquitination of DPP4 promotes synovial cell proliferation and invasion, exacerbating synovitis in RA	[14]
	TRAF6	MMP	Positive regulatory factors of MMP	Inhibition of TRAF6 can reduce collagen-induced bone loss and MMP expression levels in RA rats	[28]
	ZNRF3	β-catenin, IκBα	Positive regulatory factors of the Wnt signaling pathway and NF-κ B signaling pathway	ZNRF3 regulates collagen-induced arthritis through the NF-kB and Wnt pathways	[26]
	STUB1	p62	STUB1 is a ubiquitination positive regulator of p62	STUB1 promotes differentiation of Tfh cells in RA by mediating activation of mTORC1 pathway through p62 ubiquitination	[22]
	STUB1	AHR	STUB1-mediated ubiquitination of AHR ubiquitination sites	STUB1 promotes Th17/Treg cell imbalance through non-degradable ubiquitination of aromatic hydrocarbon receptors (AHR)	[61]
	RNF13	LAMP-1	RNF13 mediates the K48 ubiquitination of LAMP-1 at residue K128 to achieve proteasomal degradation	RNF13 promotes the TLR signaling pathway and participates in the pathogenesis of RA by mediating K48-linked polyubiquitination and subsequent degradation at the LAMP-1 K128 site	[23]
	Synoviolin	IRE1	SYVN1 catalyzes the ubiquitination of IRE1	Synoviolin promotes IRE1 ubiquitination and degradation of synovial fibroblasts in CIA mice	[29]
DUBs	USP2	TRAF2	Promote the removal of ubiquitinated chains from TRAF2 and enhance its stability	USP2 promotes the proliferation and inflammation of fibroblast-like synovial cells in rheumatoid arthritis through deubiquitination of TRAF2	[31]
	USP5	TRAF6	Positive regulatory factors of NF-κB signaling pathway	USP5 interacts with tumor necrosis factor receptor associated factor 6 (TRAF6) and removes its K48 linked polyubiquitination chain, thereby stabilizing TRAF6	[30]
Other ubiquitination-related factors	UBD	p-p38	Activation factors of the p38 MAPK pathway	UBD may mediate the activation of p38 MAPK, thereby promoting the proliferation of RA-FLS and ultimately promoting the progression of RA	[32]
	SUMO-1	Rac1	Positive regulatory factors of Rac1/PAK1 pathway	SUMO-1 mediated SUMOylation controls Rac1 activation and regulates downstream PAK1 activity. Inhibition of Rac1 or PAK1 also reduced the migration and invasion of RA-FLS	[33]
	SUMO-1	PML	SUMO-1 is involved in the formation of protein nucleosomes (NBs) in promyelocytic leukemia (PML)	SUMO-1 modified nuclear PML protein regulates Fas-induced apoptosis of rheumatoid arthritis synovial fibroblasts	[68]
	SUMO-2/3	MMP	Selective control of TNF-mediated MMP expression through NF-B pathway	SUMO-2 contributes to the specific activation of RA-FLS	[34]
	SAE1/UBA2	PKM2	Knockdown of SAE1 or UBA2 reduces nuclear translocation of PKM2	The increase of synovial SAE1/UBA2 may lead to synovial glycolysis and joint inflammation in RA	[66]

### 5.2. Synoviolin/Hrd1 and arthropathy

Using immune screening with anti-rheumatoid synovial cell antibodies, researchers have identified and cloned Synoviolin (also known as Hrd1), an E3 ubiquitin ligase highly expressed in RA synovium. Overexpression of Synoviolin in mice results in the spontaneous development of arthropathy. Conversely, Synoviolin ± mice display resistance to CIA, attributed to enhanced apoptosis of synovial cells. These findings position Synoviolin/Hrd1 as a novel pathogenic factor in inflammatory arthropathy, wherein its anti-apoptotic activity contributes to synovial hyperplasia and joint destruction [67].

### 5.3. c-MIR and inflammatory cytokine production

Gene transfer of *c-MIR* has been shown to inhibit IL-6 production by synovial fibroblasts stimulated with TNF-α or IL-1β. In addition, bone marrow-derived macrophages and DCs from *c-MIR* transgenic mice exhibit impaired production of IL-6 and TNF-α in response to LPS stimulation. Notably, this regulatory effect occurs at the post-transcriptional level, as the mRNA expression of these cytokines remains unaffected. These results uncover a previously unrecognized role of *c-MIR* in modulating inflammatory cytokine production [60].

### 5.4. UBD and RA

Elevated expression of UBD has been shown to activate phosphorylated p38 MAPK in RA-FLSs. Overexpression of UBD promotes RA-FLS viability and proliferation while suppressing apoptosis. Treatment with the p38 MAPK inhibitor SB202190 partially reverses these effects, attenuating UBD-induced proliferation and restoring apoptosis, as well as reducing the secretion of inflammatory cytokines. These findings suggest that UBD facilitates RA-FLS activation and inflammatory mediator production *via* the p38 MAPK pathway, thus contributing to RA progression. Accordingly, UBD may serve as both a therapeutic target and a prognostic biomarker in RA [32].

### 5.5. SUMO-1 and migration and invasion of RA-FLSs

Increased expression of SUMO-1 and SUMO-2 has been observed in FLSs and synovial tissues (STs) of RA patients. Silencing of SUMO-1 *via* small interfering RNA (siRNA) significantly reduces the migratory and invasive capacities of RA-FLSs, as well as the expression of matrix metalloproteinases MMP-1 and MMP-3. Further investigations suggest that SUMO-1 regulates lamellipodium formation, thereby modulating cell motility through the Rac1/PAK1 signaling pathway. These findings imply that inhibition of SUMO-1 may mitigate joint destruction in RA by suppressing the aggressive behavior of FLSs [33].

### 5.6. TRIM32 and inflammatory responses in RA-FLSs

TRIM32 expression is significantly elevated in FLSs derived from RA patients compared to those from individuals with OA. Its expression is further upregulated in a time-dependent manner upon stimulation with pro-inflammatory cytokines such as TNF-α. Overexpression of TRIM32 enhances the production of inflammatory cytokines in RA-FLSs, while knockdown of TRIM32 produces the opposite effect. Mechanistically, TRIM32 activates the NF-κB signaling pathway by interacting with TNF receptor-associated factor 2 (TRAF2) and promoting its K63-linked polyubiquitination. These results identify TRIM32 as a positive regulator of inflammatory signaling in RA-FLSs [25].

### 5.7. Mid1 and synovial cell proliferation and migration

Mid1, an E3 ubiquitin ligase, has emerged as a critical regulator of synovial activation and a potential therapeutic target in RA. Elevated expression of Mid1 has been confirmed in both human RA synovial tissues and in a CIA mouse model. Notably, Mid1-deficient mice are completely protected from CIA. Mechanistically, Mid1 promotes synovial cell proliferation and migration by facilitating the ubiquitin-mediated proteasomal degradation of DPP4. Thus, ubiquitination of DPP4 by Mid1 drives synovial hyperplasia and invasion, exacerbating synovitis in RA [14].

### 5.8. USP5 and inflammatory processes in RA-FLSs

IL-1β stimulation induces a time-dependent increase in the expression of the deubiquitinating enzyme USP5 in RA-FLSs. Overexpression of USP5 enhances the production of pro-inflammatory cytokines and activates the NF-κB signaling pathway, while USP5 knockdown attenuates cytokine release and suppresses NF-κB activation. USP5 also interacts with TRAF6 and removes its K48-linked polyubiquitin chains, thereby stabilizing TRAF6. These findings suggest that USP5 promotes inflammatory responses in RA-FLSs and represents a potential molecular target for RA treatment [30].

### 5.9. STUB1 and Th17/Treg imbalance

STUB1 regulates the balance between Th17 and Treg cells by modulating the non-degradative ubiquitination of aryl hydrocarbon receptor (AHR). Specifically, STUB1 promotes K63-linked ubiquitination of AHR, thereby altering its function and contributing to Th17/Treg cell imbalance. Given the crucial role of this imbalance in RA pathogenesis, STUB1 may serve as a novel therapeutic target by restoring immune homeostasis [61].

### 5.10. TRAF6 and MMP production

In RA, the expression levels of tissue inhibitor of TIMP-1 are significantly reduced, while the levels of MMP-1, MMP-13, and COX-2 are markedly increased. Silencing of TRAF6 reverses these changes. These findings highlight the role of TRAF6 in promoting joint destruction and suggest that its inhibition may help restore the balance between osteoclast and osteoblast activity, contributing to disease resolution in RA [28].

### 5.11. ZNRF3 and inflammation in RA-FLSs

Although the E3 ubiquitin ligase zinc and ring finger protein 3 (ZNRF3) is recognized as a negative regulator of the Wnt signaling pathway, its role in RA remains incompletely understood. Elevated expression of ZNRF3 has been observed in both RA-FLSs and in the CIA mouse model. Lentivirus-mediated silencing of *ZNRF3* significantly reduced RA-FLS viability and TNF-α-induced inflammatory responses. Furthermore, ZNRF3 knockdown in CIA mice attenuated knee joint damage and lowered levels of TNF-α, IL-1β, and IL-6. These effects are thought to result from crosstalk between the Wnt and NF-κB signaling pathways in RA-FLSs [26].

### 5.12. SUMO-1 and apoptosis resistance in RA-FLSs

Increased expression of SUMO-1 has been implicated in promoting resistance to Fas-mediated apoptosis in RA-FLSs. Mechanistically, SUMO-1 enhances the SUMOylation of the nuclear promyelocytic leukemia (PML) protein, facilitating the recruitment of the transcriptional repressor DAXX to PML nuclear bodies (PML-NBs). Reduced nuclear levels of the SUMO-specific protease SENP1, as observed in RA-FLSs, further reinforce this anti-apoptotic mechanism by impairing DAXX release from PML-NBs. The accumulation of DAXX in PML-NBs driven by SUMO-1 may thus contribute to FLS survival and the chronic inflammatory environment characteristic of RA [68].

### 5.13. SUMO-2/3 and TNF-mediated MMP expression

Compared with OA controls, RA tissues and RA-FLSs exhibit decreased expression levels of SUMO-2 and SUMO-3. However, in hTNF-transgenic (hTNFtg) mice, synovial tissues and FLSs display increased SUMO-2 expression. *In vitro* experiments show that TNF stimulation selectively induces SUMO-2, but not SUMO-3, expression. Genetic ablation of SUMO-2/3 leads to enhanced TNF-α- and IL-1β-induced expression of MMP-3 and MMP-13, accompanied by increased NF-κB signaling. These findings suggest that despite their structural similarity, SUMO-2 and SUMO-3 exhibit differential regulatory responses to TNF and contribute to the selective control of TNF-mediated MMP expression *via* NF-κB activation. Collectively, SUMO-2 appears to play a functional role in RA-FLS activation [34].

### 5.14. STUB1 and Tfh cell differentiation in RA

Elevated expression of STUB1 has also been observed in Tfh cells from RA patients. Upregulation of STUB1 promotes Tfh cell differentiation through a mechanism involving the K48-linked ubiquitination and subsequent degradation of the autophagy receptor p62. This degradation leads to the activation of the mTORC1 pathway. Notably, overexpression of p62 can reverse both Tfh cell differentiation and mTORC1 activation driven by STUB1. These findings suggest that STUB1 facilitates Tfh cell differentiation in RA by regulating mTORC1 signaling through p62 degradation [22].

### 5.15. RNF13 and endosomal TLR-mediated inflammatory responses

As a highly organized system, endolysosomes play a critical role in maintaining immune homeostasis [69]. Studies have revealed that the lysosomal E3 ubiquitin ligase RNF13 inhibits lysosomal maturation and promotes endosomal Toll-like receptor (TLR)-mediated inflammatory responses in macrophages. Mechanistically, RNF13 mediates K48-linked polyubiquitination of LAMP-1 at residue K128, targeting it for proteasomal degradation. Upon TLR activation, LAMP-1 facilitates lysosomal maturation, thereby accelerating lysosomal degradation of TLRs and reducing TLR signaling in macrophages [23].

### 5.16. SAE1/UBA2 and synovial glycolysis/joint inflammation in RA

Research indicates that glycolytic metabolism is particularly enhanced in the synovial tissue of RA patients, contributing to persistent synovial inflammation and joint damage [70–72]. Targeting specific synovial glycolytic enzymes or metabolic intermediates may hold therapeutic potential for RA [73]. SUMOylation, a post-translational protein modification, plays significant roles in the pathogenesis of many diseases [74]. However, the involvement of SAE1/UBA2 in regulating pathogenic FLS behavior remains poorly understood. Notably, SAE1 and UBA2 expression is elevated in FLS and synovial tissues of RA patients. siRNA-mediated knockdown of SAE1 or UBA2, combined with geldanamycin (GA) treatment, reduces glycolysis, invasive phenotypes, and inflammation. These findings suggest that increased synovial SAE1/UBA2 levels may drive synovial glycolysis and joint inflammation in RA, positioning SAE1/UBA2 as potential therapeutic targets [66].

### 5.17. Impact of SYVN1 on synovial cell growth in RA

SYVN1 expression levels correlate closely with Inositol-requiring enzyme 1 (IRE1) levels in synovial fibroblasts from CIA mice. Elevated SYVN1 expression reduces IRE1 levels, a key pro-apoptotic factor in endoplasmic reticulum (ER) stress-induced apoptosis. Further investigation reveals that SYVN1 interacts with and ubiquitinates IRE1, promoting its degradation. Inhibiting SYVN1 expression in CIA synovial fibroblasts restores IRE1 protein levels and reverses ER stress-induced resistance to apoptosis. These results indicate that SYVN1 suppresses ER stress-induced cell death by degrading IRE1, thereby promoting synovial cell hyperproliferation [29].

### 5.18. USP2 and RA

USP2 overexpression significantly exacerbates cellular proliferation and inflammatory responses, while its sustained downregulation reduces inflammatory cytokine secretion and inhibits cell proliferation. USP2 interacts with TRAF2 and promotes deubiquitination of TRAF2, enhancing its stability. These findings suggest that USP2 acts as a pro-proliferative and pro-inflammatory regulator in RA-FLS, highlighting its potential as a therapeutic target for RA [31] (Table 2 and Figure 3).

## 6. Therapies related to ubiquitination

A recent study employed high-throughput compound screening technology to identify small-molecule inhibitors targeting Synoviolin, with the goal of suppressing its auto-ubiquitination activity (Table 3). The screening was conducted using the Lead Discovery Service program’s pharmacopeia library, which includes over 4 million compounds. Through this large-scale screening approach, two distinct classes of small-molecule inhibitors—designated LS-101 and LS-102—were identified. Both compounds demonstrated effective inhibition of Synoviolin activity. Specifically, LS-102 selectively inhibits the enzymatic activity of Synoviolin, whereas LS-101 exhibits broader inhibitory effects against multiple RING-type E3 ligases. In functional assays, both LS-101 and LS-102 were found to suppress the proliferation of RA-derived synovial cells and significantly alleviate disease severity in RA mouse models. These findings highlight Synoviolin inhibition as a promising therapeutic strategy for RA [75]. Currently, LS-101 and LS-102 are undergoing preclinical development, with validation in both cellular and animal models. Although the selectivity profiles (particularly LS-102) and *in vivo* efficacy provide a solid basis for further development, additional pharmacokinetic/toxicological evaluations, as well as structural optimization of lead compounds, are required before clinical translation. Importantly, the broader activity spectrum of LS-101 as a pan-RING E3 ligase inhibition raises concerns regarding potential off-target effects and unpredictable adverse reactions, necessitating comprehensive safety assessments in subsequent studies.

**Table 3.** Therapeutics of ubiquitination-related enzymes in RA.

	Therapies	Effect	References
Synoviolin	LS-101 and LS-102	LS-102 selectively inhibits synoviolinase activity, while LS-101 inhibits multiple RING type E3 ligases. In addition, these inhibitors inhibited the proliferation of rheumatoid synovial cells and significantly reduced the severity of the disease in the RA mouse model.	[75]
USP7	morin	Morin restricts FLS migration and arthritis by intervening in USP7-Prickle1-mTORC2 signaling and FA turnover	[76]
RNF13	HCQ	Immunosuppressant hydroxychloroquine (HCQ) can increase polyubiquitination of RNF13	[23]
TLR4	Dopamine D3 receptor (D3R)	D3R alleviates inflammation of rheumatoid arthritis in mice through mTOR/AKT/AMPK-LC3-ubiquitin-TLR4 signaling axis	[77]

In another study, researchers explored the effects of combined overexpression of USP7 and treatment with morin on the ubiquitination of Prickle1 protein. Experimental results demonstrated that overexpression of USP7, achieved through plasmid transfection in PDGF-stimulated FLSs, attenuated the morin-induced ubiquitination and regulatory effects on Prickle1. These findings suggest that morin binds directly to USP7 and inhibits its deubiquitinating activity, thereby promoting the ubiquitination of Prickle1. To elucidate the role of the ‘USP7-Prickle1-mTORC2’ signaling axis in the morin-mediated suppression of FLS migration, researchers employed plasmid constructs and specific pathway inhibitors. Results showed that morin significantly reduced the phosphorylation of paxillin and FAK, as well as the internalization of integrin β1. However, USP7 overexpression reversed the inhibitory effect on integrin β1 internalization. Moreover, overexpression of Prickle1 and USP7, along with treatment with the mTORC1/2 agonist MHY1485, substantially reversed morin-induced inhibition of FLS migration. These findings collectively indicate that morin impairs FLS migration and arthritis progression by targeting the USP7-Prickle1-mTORC2 signaling pathway and disrupting focal adhesion turnover. Further molecular docking and biochemical assays revealed that morin exhibited stronger binding affinity to USP7 at catalytic domain residues His461, Met292, and Phe409, compared to structurally related deubiquitinating enzyme USP9X. This binding selectively inhibits USP7 activity. Importantly, USP7 overexpression reversed morin-mediated regulation of Prickle1 expression, further confirming that USP7 is a direct molecular target of morin. Through a combination of *in vitro* and *in vivo* experiments, the study elucidated the mechanistic basis by which morin suppresses FLS migration and mitigates arthritis progression [76]. As a natural compound, morin holds potential as a USP7-targeted therapeutic agent for RA; however, it remains at the preclinical validation stage (cellular/animal models). Major translational challenges include improving its bioavailability, reducing off-target effects (particularly against structurally related DUBs like USP9X), and establishing safe and effective dosing strategies. Moreover, the polypharmacological nature of morin necessitates the development of more selective USP7 inhibitors to minimize potential toxicities.

Additionally, studies have analyzed the expression levels of RNF13 and LAMP-1 proteins in PBMCs from healthy controls (HCs) and newly diagnosed RA patients. The results revealed increased RNF13 and decreased LAMP-1 expression in PBMCs from RA patients compared to HCs, suggesting that dysregulation of these proteins may be involved in the pathogenesis of inflammatory autoimmune diseases. Hydroxychloroquine (HCQ), a quinoline-derived heterocyclic compound, has long been used as an antimalarial agent and remains an approved disease-modifying antirheumatic drug (DMARD) for RA treatment, frequently administered in combination with other DMARDs [78]. To explore whether HCQ influences RNF13 expression in inflammatory responses, researchers examined its effects following LPS stimulation. HCQ treatment significantly enhanced the ubiquitination of RNF13 and concurrently reduced its protein levels, consistent with its known anti-inflammatory activity [23]. These findings offer novel mechanistic insights into HCQ’s therapeutic efficacy, potentially extending beyond its established pathways. However, despite these benefits, HCQ administration requires close clinical monitoring due to dose- and duration-dependent adverse effects, including retinopathy, cardiotoxicity, and myopathy [79].

Dopamine (DA), a key catecholamine neurotransmitter, has been shown to modulate immune cell activation through dopamine receptors (DRs) [80–82]. DA has been detected in inflamed synovial tissues of RA patients [83], and DA analogs have demonstrated therapeutic efficacy in both preclinical models and clinical settings [84]. Clinical studies have identified an inverse correlation between the number of D3R-positive mast cells (MCs) in RA synovial fluid and disease severity. *In vivo* and *in vitro* experiments revealed that D3R directly inhibits MC activation and the release of pro-inflammatory cytokines in response to LPS and methamphetamine (a DA analog) [85]. Using the CIA mouse model, researchers compared disease progression in wild-type and D3R-deficient DBA/1 mice. The absence of D3R exacerbated disease severity, suggesting a protective role for D3R. Mechanistically, D3R suppresses mast cell activation *via* a TLR4-dependent pathway by promoting LC3 lipidation and enhancing the degradation of ubiquitin-tagged TLR4. This process involves inhibition of mTOR and AKT phosphorylation, activation of AMPK signaling, and subsequent autophagy-mediated clearance of TLR4. These results demonstrate that D3R attenuates inflammation in RA through the mTOR/AKT/AMPK-LC3-ubiquitin-TLR4 axis. Collectively, these findings reveal a novel immunoregulatory role for D3R in controlling mast cell-driven inflammation and expand our understanding of RA pathogenesis [77]. While preclinical data strongly support mast cell D3R as a potential therapeutic target, clinical translation remains challenging. Although clinical correlations exist between D3R expression and RA severity, and DA analogs show preliminary promise, selective targeting of mast cell D3R to modulate TLR4 ubiquitination has yet to reach clinical evaluation. Moreover, the long-term immunological impact of modulating mast cell activation and autophagy-ubiquitination pathways must be carefully assessed to avoid impairing immune homeostasis (Table 3).

## 7. Current research limitations and future directions

Despite the promising role of ubiquitination regulatory networks in elucidating RA pathogenesis and informing targeted therapeutic strategies, several critical limitations and unresolved issues remain, warranting further investigation. First, significant discrepancies between experimental models and clinical populations present a major translational barrier. Most current studies predominantly rely on murine models of arthritis or *in vitro* cultures of RA-FLSs. However, species-specific differences, patient heterogeneity, and the confounding effects of clinical interventions (e.g. DMARDs/biologics) may limit the clinical applicability of preclinical findings. Second, challenges in achieving specificity within the ubiquitination system remain unresolved. Many ubiquitination-related enzymes, such as TRAF6 and USP5, exhibit poly-substrate activity, and their involvement in multiple signaling pathways raises concerns regarding off-target effects. Moreover, the functional consequences of different ubiquitin chain linkages (e.g. K48, K63, M1) are not yet fully understood, and redundancy among enzyme families (e.g. E3 ligases) may enable compensatory regulatory mechanisms, complicating therapeutic targeting. Third, targeted therapeutic approaches face ongoing safety concerns. Issues such as limited selectivity of small-molecule inhibitors (e.g. the broad E3 ligase inhibition profile of LS-101), cumulative toxicity resulting from disruption of disrupting ubiquitin-proteasome system (UPS) homeostasis (neuro/metabolic dysfunction), and increased risks of infection or malignancy due to immunosuppression underscore the need for rigorous benefit–risk evaluation in drug development.

This review consolidates the current understanding of the role of ubiquitination modifications in RA pathogenesis and highlights persistent challenges, including discrepancies between preclinical models and clinical populations, targeting specificity limitations, and unresolved safety concerns. Despite substantial progress, multiple critical areas require further exploration. Future research may benefit from the following directions: Elucidating the specific mechanisms of ubiquitination-related enzymes in RA: While several enzymes have been implicated in RA and correlated with disease activity and cytokine expression, their precise molecular mechanisms remain poorly defined. Future studies should investigate how these enzymes regulate key biological processes—including inflammation, immune responses, and cell proliferation—and delineate the signaling pathways they modulate. Developing targeted therapies against specific ubiquitination enzymes: Given the pivotal role of ubiquitination-related enzymes in RA, the development of selective modulators (inhibitors or agonists) holds great clinical potential. Research should focus on high-throughput screening, structure-activity relationship (SAR) optimization, and rigorous preclinical assessment of efficacy and safety. Evaluating combination strategies involving ubiquitination-related interventions: Due to the multifactorial nature of RA, single-agent therapies may be insufficient. Future studies should assess the therapeutic potential of combining ubiquitination-targeted approaches with other modalities, such as immunosuppressants, biologics, or traditional medicines, to develop more comprehensive and effective treatment regimens. Investigating the role of ubiquitination in RA-associated comorbidities: RA is often accompanied by comorbid conditions such as interstitial lung disease, cardiovascular disease and osteoporosis. Further research is needed to determine how ubiquitination modifications contribute to the pathogenesis of these comorbidities and to explore their interplay with RA, thereby informing the development of more personalized therapeutic strategies. Additionally, research must elucidate the dynamic evolution of ubiquitination networks across RA stages (e.g. early immune initiation *vs.* late structural damage) to uncover their temporal regulatory principles.

Among the numerous potential ubiquitination-related targets in RA, the following candidates warrant prioritization based on the strength of current evidence, mechanistic clarity, and therapeutic feasibility: firstly, A20 (TNFAIP3) serves as a critical negative feedback regulator of proinflammatory signaling, particularly the NF-κB pathway (Section 5.1). Strong experimental evidence demonstrates that myeloid-specific deletion of A20 leads to spontaneous, erosive arthritis that closely resembles human RA. Moreover, A20 expression is significantly reduced in PBMCs of RA patients and negatively correlates with CRP/anti-CCP levels (Sections 3 and 4) [13,21]. Therapeutic strategies that enhance A20 expression/activity (e.g. gene therapy, small-molecule activators) hold great promise in suppressing innate immune overactivation and downstream inflammation, effectively acting as a ‘brake’ on inflammatory cascades. The cell-type specificity of A20 (e.g. in the myeloid lineage) further supports its potential for precision targeting. Future research should focus on developing safe and efficacious A20-augmenting interventions and assessing their therapeutic efficacy across diverse RA subtypes. Second, Synoviolin (also known as Hrd1) has been clearly validated as a pathogenic driver in RA. Overexpression of Synoviolin promotes synoviocyte survival by inhibiting apoptosis, contributing to synovial hyperplasia and joint damage (Section 5.2) [67]. Critically, small-molecule Synoviolin inhibitors (e.g. LS-102) have shown efficacy in reducing synoviocyte proliferation and attenuating disease severity in RA animal models [75], thereby confirming its therapeutic viability. Targeting Synoviolin offers a direct approach to modulating the pathological behavior of synovial fibroblasts by counteracting their anti-apoptotic and invasive phenotypes. Future priorities include the optimization of highly selective Synoviolin inhibitors, comprehensive pharmacodynamic/safety evaluations, and evaluation of potential synergy with conventional DMARDs. Third, STUB1 has emerged as a key regulator of immune cell homeostasis, particularly in modulating the Th17/Treg balance and Tfh cell differentiation—key processes underlying RA autoimmunity (Sections 5.9 and 5.14) [22,61]. Dysregulated STUB1 expression in RA patient T cells (particularly Th17/Treg subsets) promotes the production of proinflammatory cytokines (e.g. IL-17A, IL-6, TNF-α) and suppresses anti-inflammatory mediators (e.g. IL-10, TGF-β), strongly correlating with disease activity (Sections 3 and 4). Therapeutic modulation of STUB1 (e.g. *via* inhibitors) could restore immune balance by correcting Th17/Treg and Tfh imbalances, offering an upstream strategy to modulate RA pathogenesis at its autoimmune roots. Given the centrality of immune modulation in long-term RA control, future efforts should focus on elucidating STUB1’s cell-specific roles and developing selective pharmacological regulators. In summary, A20, Synoviolin, and STUB1 represent distinct yet mechanistically critical nodes in RA pathogenesis—spanning inflammatory regulation, synoviocyte survival/invasion, and immune cell dysregulation. Each target is supported by robust mechanistic insights and proof-of-concept therapeutic data. Prioritizing in-depth exploration of these pathways may accelerate the development of novel, mechanism-based therapies with the potential to transform RA treatment paradigms.

## 8. Conclusion

This study provides a comprehensive analysis of current research on ubiquitination modifications in RA, reinforcing their mechanistic involvement in RA pathogenesis and evaluating their potential as therapeutic targets. Accumulating evidence indicates that multiple ubiquitination-related enzymes are abnormally expressed in RA and show strong associations with disease activity and proinflammatory cytokine levels. These enzymes play pivotal roles in regulating key pathological processes, including inflammatory signaling, immune responses, and synovial cell proliferation. Collectively, these findings consolidate the conceptual framework positioning the ubiquitination system as a core pathological axis in RA. Furthermore, they offer valuable insights and experimental evidence supporting the development of novel therapeutic strategies targeting specific components of the ubiquitination machinery.

## Data Availability

Data sharing is not applicable to this article as no new data were created or analyzed in this study.
